# Successful Management of Hepatic Abscess From Ingested Foreign Body: A Case Report

**DOI:** 10.1155/cris/6987968

**Published:** 2026-06-24

**Authors:** Samin Rostamzadeh, Fatemeh Shahrahmani, Davood Salehi, Mohammadreza Mohammad Hosseiniazar

**Affiliations:** ^1^ Department of General Surgery, School of Medicine, Urmia University of Medical Science, Urmia, Iran, umsu.ac.ir; ^2^ Faculty of Medicine, Mashhad University of Medical Sciences, Mashhad, Iran, mums.ac.ir; ^3^ Department of Internal Medicine, School of Medicine, Urmia University of Medical Science, Urmia, Iran, umsu.ac.ir

**Keywords:** case report, foreign body, gastrointestinal perforation, hepatic abscess

## Abstract

Hepatic abscess secondary to foreign body ingestion is a rare but potentially life‐threatening condition. Although most ingested foreign bodies pass harmlessly through the gastrointestinal tract, sharp objects may perforate the gastric or intestinal walls, migrate into adjacent organs, and lead to abscess formation. This report describes a 22‐year‐old female with a 10‐year history of systemic lupus erythematosus (SLE) who presented with persistent epigastric discomfort, nausea, and vomiting for 2 weeks. Initial evaluations, including laboratory tests and abdominal ultrasonography, were unremarkable. Subsequent endoscopy revealed a foreign body lodged in the distal stomach that could not be removed endoscopically. A contrast‐enhanced computed tomography (CT) scan demonstrated a 7 cm × 5.6 cm abscess in the left hepatic lobe. The patient underwent laparotomy, which confirmed a toothpick penetrating the gastric wall and lodging in the liver. The abscess was drained, and gastrorrhaphy was performed. The patient recovered uneventfully following antibiotic therapy and was discharged in stable condition, with no recurrence during follow‐up. Foreign body–induced hepatic abscesses are uncommon, and diagnosis can be challenging because classical signs of infection may be absent or attenuated. Endoscopy remains the first‐line diagnostic and therapeutic approach; however, surgical intervention is required when the foreign body is embedded or complications occur. Clinicians should maintain a high index of suspicion in patients with persistent or unexplained abdominal symptoms, particularly in immunocompromised individuals.

## 1. Introduction

The accidental ingestion of foreign bodies is relatively common, particularly among children and individuals with certain mental health conditions [1, 2]. While most ingested objects pass harmlessly through the gastrointestinal tract, a small proportion may deviate from the expected course and lead to serious complications [3]. Sharp objects, in particular, can perforate the gastrointestinal tract and migrate into adjacent organs [4]. In rare cases, such migration results in hepatic abscess formation [5].

These abscesses often present with nonspecific symptoms, including fever, abdominal pain, nausea, and fatigue, mimicking a wide range of other conditions [6]. Although a history of foreign body ingestion may raise clinical suspicion, many patients are unaware of the ingestion event. Persistent or unexplained symptoms that do not respond to initial treatment should prompt further evaluation [1]. Diagnosis remains challenging due to the nonspecific presentation and the rarity of the condition. Imaging modalities, particularly ultrasound and computed tomography (CT), play a crucial role in identifying foreign bodies and associated complications [7].

Here, we report a case of hepatic abscess caused by a foreign body that penetrated the distal gastric antrum.

## 2. Case Presentation

A 22‐year‐old female presented with epigastric discomfort, nausea, and vomiting for 2 weeks. She described persistent epigastric pain that was not influenced by food intake or changes in body position but radiated to the shoulder. The pain was partially relieved by over‐the‐counter analgesics. She denied fever, chills, melena, hematemesis, or changes in bowel habits. Initial outpatient evaluation, including laboratory tests and abdominal ultrasonography, revealed no significant abnormalities.

Her medical history was notable for a 10‐year history of systemic lupus erythematosus (SLE), as well as hypertension and depression. Her medications included prednisolone (5 mg daily), hydroxychloroquine, mycophenolate mofetil (1 g twice daily), losartan, and fluoxetine. She had also been taking rivaroxaban for several years following a pulmonary embolism and had previously undergone pericardiocentesis for cardiac tamponade, with no evidence of malignancy.

On admission, the patient was awake and oriented, with stable vital signs. Physical examination revealed mild epigastric tenderness without signs of peritonitis, such as rebound tenderness or guarding. No organomegaly was noted. Laboratory findings showed a white blood cell count of 9600/µL, hemoglobin of 11.6 g/dL, C‐reactive protein of 19 mg/L, and erythrocyte sedimentation rate of 94 mm/h, along with mild proteinuria. Other laboratory parameters, including liver function tests, amylase, lipase, and coagulation studies, were within normal limits. Electrocardiography demonstrated a normal sinus rhythm. Chest radiography was unremarkable, with no evidence of free air. Repeat abdominal ultrasonography was also normal. Given her history of pulmonary thromboembolism and cardiac tamponade, a cardiology consultation was obtained; echocardiography revealed no abnormalities.

Due to persistent symptoms, upper gastrointestinal endoscopy was performed with a presumptive diagnosis of peptic ulcer disease. Endoscopy revealed an impacted foreign body in the distal stomach that could not be removed endoscopically. Given concern for possible tissue involvement, a contrast‐enhanced abdominal CT scan was performed. The CT scan demonstrated a 7 cm × 5.6 cm multiloculated hypodense lesion in segments II and III of the left hepatic lobe, consistent with a hepatic abscess. The lesion was located in close proximity to the gastric wall. The foreign body was not directly visualized, likely due to its radiolucency; however, the anatomical relationship suggested transgastric penetration (Figure 1).

**Figure 1:**
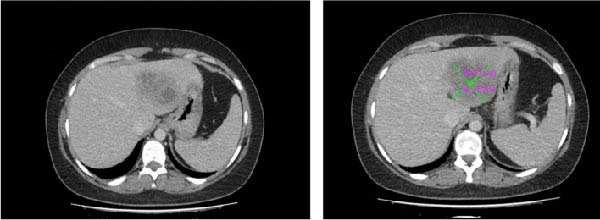
Contrast‐enhanced CT scan demonstrating a multiloculated cystic lesion measuring 7 cm × 5.6 cm in segments II and III of the liver.

The patient subsequently underwent a laparotomy. A toothpick was identified and successfully removed, followed by gastrorrhaphy. The hepatic abscess was unroofed and drained (Figure 2). An intraoperative sample was obtained, and histopathological evaluation confirmed abscess formation within the liver parenchyma. Microbiological cultures revealed polymicrobial growth, including Streptococcus species and *Escherichia coli*.

**Figure 2:**
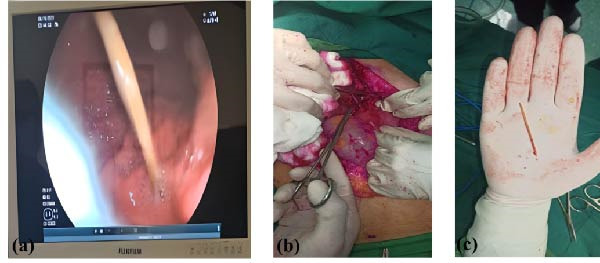
(a) Endoscopic image showing a foreign body lodged in the stomach. (b) Intraoperative view of the foreign body penetrating the stomach and extending into the liver. (c) The removed foreign body, identified as a toothpick.

Postoperatively, the patient received intravenous ceftriaxone and metronidazole for 48 h as empiric therapy; based on culture results, this regimen was continued for a total of 10 days. She was then transitioned to oral ciprofloxacin and metronidazole for an additional 4 weeks. The patient remained unaware of the foreign body ingestion throughout the hospitalization. She recovered uneventfully and was discharged in a stable condition. Follow‐up at 2 and 6 weeks demonstrated complete clinical recovery, with no evidence of recurrence.

## 3. Discussion

Gastrointestinal perforation due to ingested foreign bodies occurs in less than 1% of cases and most commonly involves the stomach and duodenum. Hepatic abscess formation secondary to foreign body migration is even rarer [8]. Toothpicks and fish bones are the most frequently reported causative agents. Reported risk factors include psychiatric disorders, substance abuse, dental prostheses, and conditions associated with diminished sensation or altered swallowing mechanisms [9, 10]. In the present case, the patient’s history of depression may have contributed to the ingestion event. Common symptoms include dysphagia, abdominal pain, and vomiting [4]. Our patient presented with persistent epigastric pain, nausea, and vomiting, consistent with foreign body ingestion, although she was unaware of the event.

The diagnosis of hepatic abscess caused by foreign body migration is particularly challenging due to the lack of specific clinical features and difficulty in identifying the foreign body. In cases of hepatic abscess without typical etiologies, such as biliary disease or sepsis, foreign body ingestion should be considered [10, 11].

Management depends on the location of the foreign body, the presence of complications, and the patient’s clinical condition [4]. Endoscopy is the first‐line diagnostic and therapeutic approach when the foreign body is accessible within the gastrointestinal tract [12]. However, it may fail if the object is embedded or has migrated. A prolonged interval between ingestion and intervention, as well as the sharpness of the object, is associated with an increased risk of complications and unsuccessful endoscopic removal [9]. In this case, endoscopy successfully identified the impacted foreign body but failed to remove it due to its embedded position and suspected tissue involvement, necessitating surgical intervention. Percutaneous drainage may be appropriate for uncomplicated abscesses without retained foreign bodies, whereas laparoscopic surgery can be considered in selected cases. Open surgery remains the preferred approach for large abscesses, deeply embedded foreign bodies, or when gastrointestinal repair is required [13]. In the present case, laparotomy was chosen due to the size and location of the abscess, as well as the presence of a penetrating foreign body. Additionally, the need for simultaneous gastric repair (gastrorrhaphy) further supported an open surgical approach.

These findings are consistent with previous reports. Alzahrani et al. [14] described a 54‐year‐old female with chronic epigastric pain, fever, and elevated liver enzymes; CT imaging revealed a foreign body penetrating the gastric pylorus and extending into the left hepatic lobe with associated portal vein thrombosis. The patient underwent laparoscopic removal of a 4 cm toothpick. Similarly, Kalkum et al. [15] reported a 50‐year‐old woman with upper abdominal pain, fever, and elevated liver enzymes; imaging identified a liver abscess caused by a toothpick, which was successfully removed laparoscopically with repair of the gastric wall defect. Kishawi et al. [16] described a 72‐year‐old man presenting with right upper quadrant pain, fever, and chills; imaging revealed a foreign body penetrating the posterior gastric wall into a branch of the left portal vein. The patient underwent exploratory laparotomy with successful retrieval of the toothpick and fistula ligation. Abu‐Wasel et al. [17] reported a 45‐year‐old woman who developed sepsis from a liver abscess caused by a wooden foreign body; after prolonged antibiotic therapy, the object was removed laparoscopically, resulting in resolution of the abscess.

Previous reports typically describe patients presenting with fever and leukocytosis [10]. In contrast, our patient lacked these classical inflammatory signs. This atypical presentation is likely related to her immunosuppressive therapy, including prednisolone and mycophenolate mofetil, which can attenuate systemic inflammatory responses and complicate the diagnosis. Corticosteroids suppress inflammation by inhibiting cytokine production and reducing leukocyte activation and migration, while mycophenolate mofetil impairs lymphocyte proliferation. Together, these effects may mask clinical and laboratory signs of infection, potentially delaying the diagnosis [18]. Similar challenges have been reported in immunocompromised patients [19]. Additionally, our patient was relatively young compared to most reported cases, which more commonly involve older individuals with identifiable risk factors. The large abscess in the left hepatic lobe is consistent with transgastric migration, as described in previous studies [10]. However, the absence of systemic inflammatory signs contributed to the diagnostic uncertainty. Therefore, clinicians should maintain a high index of suspicion when evaluating persistent or unexplained symptoms, particularly in immunocompromised patients.

The rarity of this condition underscores the importance of early recognition and appropriate management to prevent serious complications. Increased clinical awareness is crucial for early detection, particularly in patients with atypical presentations.

## Funding

No funding was received for this research.

## Ethics Statement

According to institutional guidelines, ethical approval was not required for this type of case report.

## Consent

Written informed consent was obtained from the patient for the publication of this case report and any accompanying images.

## Conflicts of Interest

The authors declare no conflicts of interest.

## Data Availability

The data that support the findings of this study are available from the corresponding author upon reasonable request.
